# Quality and reliability of semaglutide-related health information on Chinese short-video platforms: a cross-sectional study of Bilibili and TikTok

**DOI:** 10.3389/fpubh.2026.1826485

**Published:** 2026-08-05

**Authors:** Hualin Li, Fanglin Peng, Haidong Wang

**Affiliations:** 1 Department of Pharmacy, The First People's Hospital of Lianyungang/The Affiliated Lianyungang Hospital of Xuzhou Medical University/Nanjing Medical University Kangda College First Affiliated Hospital, Lianyungang, China; 2 Department of Pharmacy, Yangxin People’s Hospital, Huangshi, Hubei, China

**Keywords:** Bilibili, DISCERN, semaglutide, short video, TikTok

## Abstract

**Background:**

Semaglutide, a glucagon-like peptide-1 receptor agonist originally developed for glycemic control, has recently gained widespread attention for its weight-loss effects. With the rapid rise of short-video platforms, these media have become important channels for disseminating health information. However, the quality, reliability, and underlying determinants of semaglutide-related content on such platforms have not been systematically characterized.

**Methods:**

A cross-sectional content analysis was conducted on semaglutide-related videos from Bilibili and TikTok. Videos were collected on February 4, 2026. Two independent reviewers evaluated the quality of each video using the Global Quality Scale (GQS), modified DISCERN (mDISCERN), and Journal of the American Medical Association (JAMA) benchmark criteria. Multivariable regression analyses were performed to identify factors associated with information quality.

**Results:**

A total of 225 videos were included (107 from Bilibili and 118 from TikTok). Most videos focused on weight loss (82.2%), while only 2.7% primarily discussed hypoglycemic effects. The overall median scores were 4.0 (3.0–4.0) for GQS, 4.0 (3.0–5.0) for mDISCERN, and 2.0 (1.0–3.0) for JAMA. TikTok videos had significantly higher JAMA scores than Bilibili videos (median 3 vs. 1, *p* < 0.001), while GQS and mDISCERN scores were comparable between platforms. In multivariable analyses, videos uploaded by medical professionals were consistently associated with higher quality scores across all three instruments, whereas commercial videos were associated with lower mDISCERN and GQS scores. Higher numbers of likes were independently associated with lower mDISCERN and GQS scores overall, and a higher number of comments on TikTok was similarly associated with lower quality scores, whereas a higher number of shares on Bilibili was positively associated with mDISCERN and JAMA scores.

**Conclusion:**

Semaglutide-related videos on Chinese short-video platforms demonstrated relatively high subjective quality and usability (mDISCERN and GQS), but markedly lower adherence to formal transparency benchmarks (JAMA), with substantial variability driven primarily by uploader professional background. Content produced by medical professionals tends to provide more reliable information, while engagement metrics do not consistently reflect information quality.

## 1 Introduction

Semaglutide is a glucagon-like peptide-1 receptor agonist (GLP-1 RA) originally developed for the treatment of type 2 diabetes mellitus. Semaglutide exerts its therapeutic effects by enhancing glucose-dependent insulin secretion, delaying gastric emptying, and suppressing appetite, collectively contributing to improved glycemic control and cardiovascular outcomes in individuals with type 2 diabetes (1, 2). The robustness of these effects is supported by extensive evidence from large randomized controlled trials (RCTs) demonstrating superior HbA1c reduction and weight loss, as well as favorable cardiovascular safety profiles compared with various comparators (1, 3). Based on these data, semaglutide has subsequently been approved for chronic weight management in individuals with obesity or overweight accompanied by weight-related comorbidities, marking its transition from a glucose-lowering agent to a multidimensional anti-obesity therapy (1, 4).

In China, semaglutide was first approved for the treatment of type 2 diabetes mellitus and later received regulatory approval for weight management. With the expansion of its indications, public interest in semaglutide has increased substantially, extending beyond patients with diabetes to the general population seeking weight loss. This growing attention has coincided with the rapid rise of short-form video platforms as major channels for health information dissemination in China.

Short-video platforms have emerged as increasingly influential channels for health information dissemination in China. Platforms such as Bilibili, TikTok (Douyin in China), and others host large volumes of medical and health-related content, offering high accessibility, engaging visuals, and rapid dissemination relative to traditional health education formats. This combination enables health information to reach broad audiences in a short time (5). However, the brevity of content, algorithm-driven recommendations, and platform design can promote simplified or sensationalized messaging, potentially compromising information accuracy, balance, and overall quality (6). Given the observed variability in content quality across health topics on Chinese short-video platforms, there is a critical need for ongoing content quality evaluation and for strategies to improve reliability and public health literacy on short-video platforms. Moreover, evidence from studies focusing on obesity, diabetes, dermatology, and other health topics on Chinese short-video platforms indicates that content produced by medical professionals tends to be of higher quality, but engagement is frequently driven by factors beyond quality, including creator popularity and entertainment value (7). These patterns underscore the necessity for platform-level governance, content certification, and algorithmic incentives that prioritize evidence-based health information to bolster public health outcomes (8, 9).

To date, only a small number of studies have examined the quality of semaglutide-related content on Chinese short-video platforms, with existing work relying primarily on bivariate comparisons of quality scores by content source and type. Whether information quality is independently associated with uploader characteristics, commercial intent, and platform-specific engagement patterns after adjusting for potential confounders remains insufficiently understood. Therefore, this study aimed to assess the quality of semaglutide-related videos on two major Chinese short-video platforms using validated evaluation tools and compare video characteristics and quality between platforms, identifying factors associated with information quality overall and within each platform.

## 2 Methods

### 2.1 Study design

This cross-sectional content analysis evaluated the quality and reliability of short videos related to semaglutide on two major Chinese short-video platforms: Bilibili and TikTok (Douyin in China).

### 2.2 Video retrieval and selection

Videos were retrieved on February 4, 2026, using the Chinese keyword equivalent of “semaglutide” (司美格鲁肽) on both platforms. Searches were conducted using newly registered accounts with no prior search history related to semaglutide or weight-loss topics, under each platform’s default (non-personalized) browsing condition. Search results were sorted according to each platform’s default relevance ranking. To verify the adequacy of the generic-name search strategy, we additionally conducted a supplementary search using the Chinese brand name “诺和泰” (Ozempic/Wegovy) on both platforms. This verification is described in the Limitations section.

The top 150 videos from each platform were initially screened. Videos were excluded if they were unrelated to semaglutide, duplicated, non-informational (e.g., pure entertainment), unavailable at the time of review, or lacked audiovisual content.

**Figure 1:**
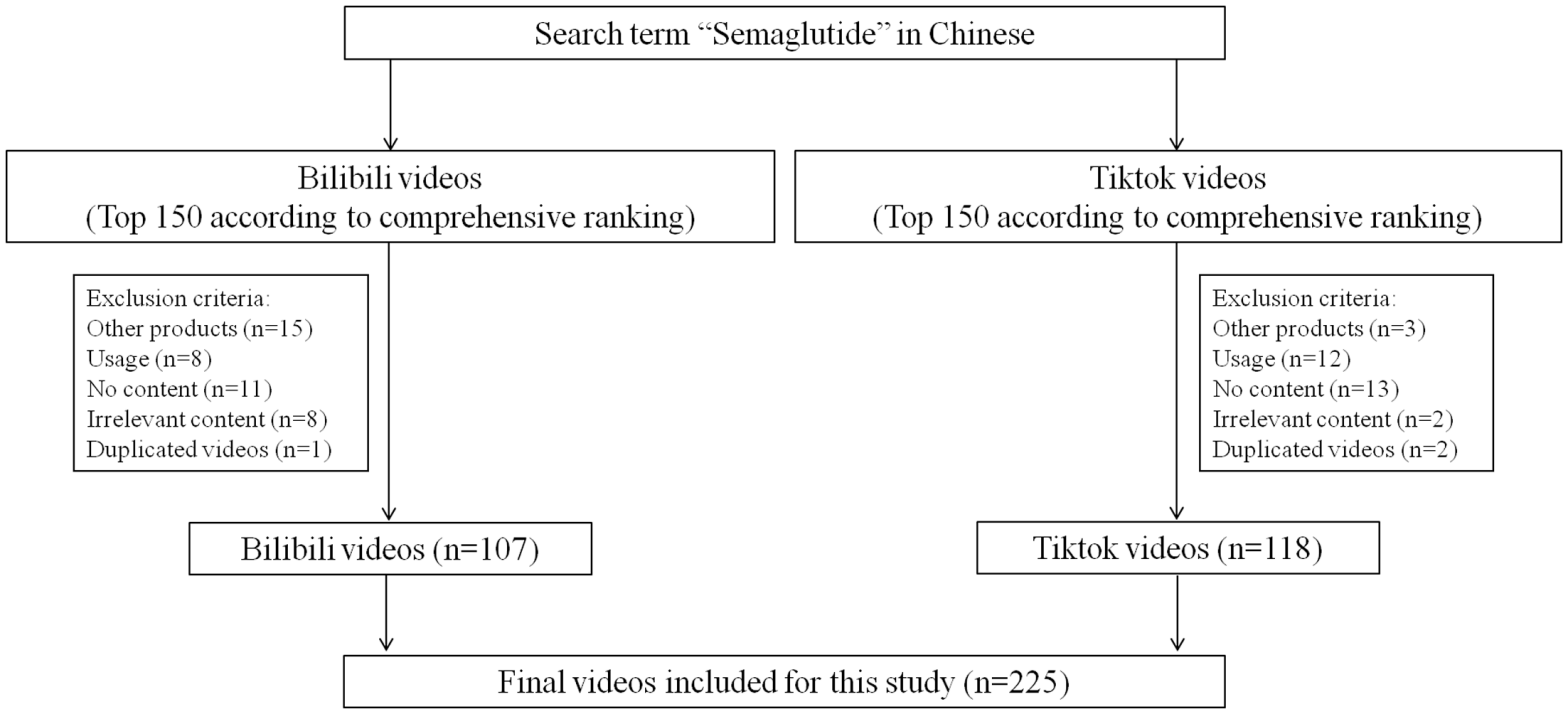
Study flowchart.

After screening, a total of 225 videos were included in the final analysis. The detailed selection process is illustrated in Figure 1.

### 2.3 Data extraction

For each eligible video, data were extracted at the time of collection across four domains: platform identity (Bilibili or TikTok); temporal and structural characteristics, including video duration (seconds) and days since publication; engagement metrics, comprising likes, favorites, and shares common to both platforms, alongside platform-specific indicators available only on one platform (comments on TikTok; total views, danmaku, and coins on Bilibili); and content-level variables, including content type (weight loss, hypoglycemic, combined, or other), uploader type (medical worker, non-medical individual, or corporate account), and commercial intent (presence or absence). All engagement metrics were recorded as displayed on the platform on the date of retrieval.

### 2.4 Video quality assessment

Video quality and reliability were evaluated using three validated instruments:

Modified DISCERN (mDISCERN), consisting of five items assessing clarity, indication description, discussion of risks, balance of information, and use of supporting evidence (score range: 0–5) (10);

Global Quality Scale (GQS), a 5-point Likert scale evaluating overall educational quality and usefulness (11);

JAMA benchmark criteria, assessing authorship, attribution, disclosure, and currency (score range: 0–4) (12).

Two reviewers independently evaluated all videos using three validated instruments [the Global Quality Scale (GQS), the modified DISCERN (mDISCERN), and the Journal of the American Medical Association (JAMA) benchmark criteria]. Disagreements were resolved through discussion until consensus was reached. Prior to independent coding, both reviewers independently scored a calibration subset of 20 videos per platform (40 videos in total) selected from the videos retained after exclusion criteria were applied, in order to assess inter-rater reliability. Cohen’s kappa for categorical variables ranged from 0.59 to 1.00, and intraclass correlation coefficients for continuous quality scores ranged from 0.83 to 0.94, indicating substantial to excellent agreement. Detailed scoring criteria for these instruments are provided in Supplementary Tables S1–S3.

### 2.5 Statistical analysis

Continuous variables were expressed as medians with interquartile ranges (*Q*1, *Q*3), and categorical variables as counts and percentages. Differences between platforms were assessed using the Mann–Whitney *U* test or chi-square test, as appropriate.

Multivariable linear regression analyses were conducted to identify factors associated with mDISCERN, GQS, and JAMA scores in the overall sample and stratified by platform. Engagement variables were log-transformed using the natural logarithm [log(*x* + 1)] to reduce right-skewed distributions (13, 14). Given evidence of severe multicollinearity among engagement metrics [variance inflation factor (VIF) > 10 for several indicators within each platform; Supplementary Table S4], each engagement variable was entered individually in separate regression models rather than simultaneously.

As sensitivity analysis to account for the bounded and ordinal nature of the mDISCERN, GQS, and JAMA scores, ordinal logistic regression was additionally performed, with each engagement metric entered individually as in the linear models above. Because the Corporate account category was sparsely represented on both platforms (*n* = 2 on Bilibili; *n* = 5 on TikTok), this category was combined with the Non-medical worker category into a single ‘Non-medical/Corporate’ group to avoid estimation instability. On TikTok, the relationship between uploader type and JAMA score exhibited quasi-complete separation (non-medical/corporate uploaders were concentrated almost exclusively at low JAMA scores, with no overlap with medical-worker uploaders at higher scores), precluding stable coefficient estimation; results for this platform–outcome combination are therefore not reported, and JAMA findings for TikTok instead rely on the linear regression and descriptive results reported above. Full results of the ordinal regression sensitivity analysis are provided in Supplementary Table S5.

Data processing and analysis were performed using R version 4.2.3 (R Foundation for Statistical Computing, Vienna, Austria) and Zstats v1.0.^1^ All statistical analyses were performed using two-sided tests, with *p* < 0.05 considered statistically significant.

## 3 Results

### 3.1 Video selection and characteristics

A total of 225 semaglutide-related videos were included in the final analysis following the screening process (Figure 1), comprising 107 videos from Bilibili and 118 videos from TikTok. The characteristics of the included videos are summarized in Table 1.

**Table 1:** Characteristics of semaglutide-related videos on Bilibili and Tiktok.

Variables	Total (*n* = 225)	Bilibili (*n* = 107)	Tiktok (*n* = 118)	*p*
Median (1st quartile, 3rd quartile)				
Likes	242 (72, 1,241)	151 (46, 788)	374.50 (103.25, 1,685)	<0.001*
Favorites	97 (23, 483)	83 (24.50, 378)	118.50 (23, 562)	0.295
Shares	105 (22, 598)	73 (14.50, 314)	138.50 (28, 991)	0.006*
Days since publish	160 (44, 581)	568 (139, 789)	72.50 (31, 172)	<0.001*
Video duration (s)	134 (84, 293)	258 (126, 533)	99.50 (66, 148)	<0.001*
Comments			104 (18, 490)	
Total views		18,000 (4,371, 59,500)		
Total danmaku		7 (1, 53)		
Coins		26 (6, 142)		

Data are presented as median (*Q*1–*Q*3).

Statistical analysis: Differences between Bilibili and TikTok were assessed using the Mann–Whitney *U* test for continuous variables.

*p-value <0.05.

Overall, the median number of likes was 242 [interquartile range (IQR): 72–1,241], and the median number of shares was 105 (IQR: 22–598). Compared with Bilibili, TikTok videos had significantly higher numbers of likes (median: 374.5 vs. 151, *p* < 0.001) and shares (median: 138.5 vs. 73, *p* = 0.006). In contrast, videos on Bilibili had a significantly longer time since publication (median: 568 vs. 72.5 days, *p* < 0.001) and longer video duration (median: 258 vs. 99.5 s, *p* < 0.001).

Platform-specific engagement indicators were also observed. Comments were available only for TikTok videos, whereas total views, danmaku counts, and coins were unique to Bilibili.

### 3.2 Comparison of content characteristics and quality scores between platforms

The distribution of content characteristics and quality scores between Bilibili and TikTok is presented in Table 2. The majority of videos focused on weight loss-related content (82.22%), with no significant difference in content type distribution between platforms (*p* = 0.399).

**Table 2:** Characteristics and information quality scores of semaglutide-related videos on Bilibili and TikTok.

Variables	Total (*n* = 225)	Bilibili (*n* = 107)	Tiktok (*n* = 118)	*p*
Content type, *n* (%)				0.399
Loss weight	185 (82.22)	86 (80.37)	99 (83.90)	
Hypoglycemic	6 (2.67)	5 (4.67)	1 (0.85)	
Loss weight + hypoglycemic	15 (6.67)	7 (6.54)	8 (6.78)	
Others	19 (8.44)	9 (8.41)	10 (8.47)	
Uploader type, *n* (%)				<0.001*
Medical worker	117 (52.00)	37 (34.58)	80 (67.80)	
Non-medical worker	101 (44.89)	68 (63.55)	33 (27.97)	
Corporate account	7 (3.11)	2 (1.87)	5 (4.24)	
Commercial, *n* (%)				1
Yes	4 (1.78)	2 (1.87)	2 (1.69)	
No	221 (98.22)	105 (98.13)	116 (98.31)	
mDISCERN total scores, *M* (*Q*1, *Q*3)	4.00 (3.00, 5.00)	4.00 (3.00, 5.00)	4.00 (3.00, 5.00)	0.409
GQS total scores, *M* (*Q*1, *Q*3)	4.00 (3.00, 4.00)	4.00 (3.00, 4.00)	4.00 (3.00, 4.00)	0.764
JAMA Scores, *M* (*Q*1, *Q*3)	2.00 (1.00, 3.00)	1.00 (1.00, 2.00)	3.00 (1.00, 3.00)	<0.001*

Data are presented as *n* (%) or median (*Q*1–*Q*3).

Statistical analysis: Differences between Bilibili and TikTok were assessed using the Chi-square test for categorical variables and the Mann–Whitney *U* test for continuous variables.

mDISCERN, modified DISCERN instrument; GQS, Global Quality Scale; JAMA, Journal of the American Medical Association benchmark criteria.

*p-value <0.05.

Uploader type differed significantly between platforms (*p* < 0.001). TikTok had a higher proportion of videos uploaded by medical workers (67.80%), whereas Bilibili had a higher proportion of non-medical uploaders (63.55%). The proportion of commercial videos was low and comparable across platforms.

Regarding information quality, no significant difference was observed between platforms for GQS scores (median: 4.0 vs. 4.0, *p* = 0.764) or mDISCERN total scores (median: 4.0 vs. 4.0, *p* = 0.409). However, TikTok videos demonstrated significantly higher JAMA scores than Bilibili videos (median: 3.0 vs. 1.0, *p* < 0.001).

### 3.3 Distribution of mDISCERN scores by uploader type

**Figure 2:**
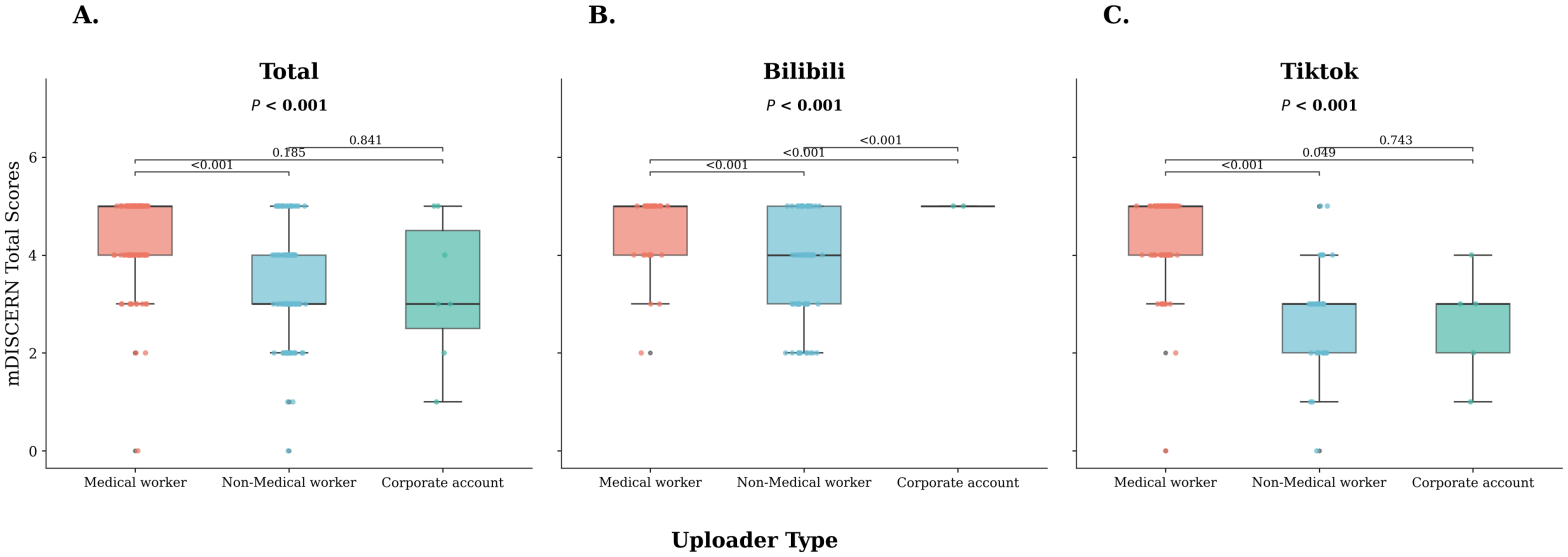
Distribution of mDISCERN scores according to uploader type. Panel **(A)** shows the results for total samples,Panel **(B)** shows the results for Bilibili, and Panel **(C)** shows the results for TikTok.

The distribution of mDISCERN scores by uploader type in the overall sample and stratified by platform is illustrated in Figure 2. Videos uploaded by medical workers consistently demonstrated higher median quality scores than those uploaded by non-medical individuals or corporate accounts.

### 3.4 Factors associated with information quality in the overall sample

**Figure 3:**
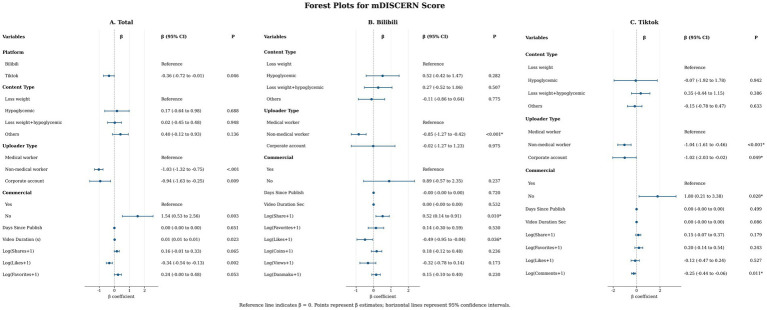
Forest plots of multivariable regression models for mDISCERN scores in the overall sample and by platform. Figure legend: Panel **(A)** shows the multivariable regression results for the overall sample. Panel **(B)** shows the results for Bilibili, and Panel **(C)** shows the results for TikTok. Points represent regression coefficients (*β*), and horizontal lines indicate 95% confidence intervals. The vertical dashed line represents *β* = 0. Reference categories are shown as “Reference”.

Multivariable linear regression analyses for mDISCERN, GQS, and JAMA scores in the overall sample are shown in Table 3 and Figure 3.

**Table 3:** Multivariable linear regression analysis of factors associated with mDISCERN score, GQS scores and JAMA scores.

Variables	mDISCERN total scores		GQS scores		JAMA scores	
	*p*	*β* (95%CI)	*p*	*β* (95%CI)	*p*	*β* (95%CI)
Platform						
Bilibili		Reference		Reference		Reference
Tiktok	0.046*	−0.36 (−0.72 ~ −0.01)	0.711	−0.05 (−0.31 ~ 0.21)	0.096	0.18 (−0.03 ~ 0.39)
Content type						
Loss weight		Reference		Reference		Reference
Hypoglycemic	0.688	0.17 (−0.64 ~ 0.98)	0.994	0.00 (−0.60 ~ 0.60)	0.216	0.31 (−0.18 ~ 0.79)
Loss weight + hypoglycemic	0.948	0.02 (−0.45 ~ 0.48)	0.830	0.04 (−0.31 ~ 0.38)	0.767	0.04 (−0.24 ~ 0.32)
Others	0.136	0.40 (−0.12 ~ 0.93)	0.392	0.17 (−0.22 ~ 0.57)	0.207	0.20 (−0.11 ~ 0.52)
Uploader type						
Medical worker		Reference		Reference		Reference
Non-medical worker	<0.001*	−1.03 (−1.32 ~ −0.75)	<0.001*	−0.74 (−0.95 ~ −0.53)	<0.001*	−1.64 (−1.81 ~ −1.47)
Corporate account	0.009*	−0.94 (−1.63 ~ −0.25)	<0.001*	−1.12 (−1.64 ~ −0.61)	<0.001*	−1.22 (−1.64 ~ −0.81)
Commercial						
Yes		Reference		Reference		Reference
No	0.003*	1.54 (0.53 ~ 2.56)	0.005*	1.09 (0.34 ~ 1.84)	0.697	0.12 (−0.49 ~ 0.73)
Log(Shares + 1)	0.065	0.16 (−0.01 ~ 0.33)	0.288	0.07 (−0.06 ~ 0.20)	0.431	0.04 (−0.06 ~ 0.14)
Log(Likes + 1)	0.002*	−0.34 (−0.54 ~ −0.13)	0.006*	−0.22 (−0.37 ~ −0.06)	0.268	−0.07 (−0.19 ~ 0.05)
Log(Favorites + 1)	0.053	0.24 (−0.00 ~ 0.48)	0.056	0.18 (−0.00 ~ 0.36)	0.517	0.05 (−0.10 ~ 0.19)
Days since publish	0.651	0.00 (−0.00 ~ 0.00)	0.764	−0.00 (−0.00 ~ 0.00)	<0.001*	−0.01 (−0.99 ~ −0.01)
Video duration (s)	0.023*	0.01 (0.01 ~ 0.01)	<0.001*	0.01 (0.01 ~ 0.01)	<0.001*	0.01 (0.01 ~ 0.01)

mDISCERN, modified DISCERN instrument; GQS, Global Quality Scale; JAMA, Journal of the American Medical Association benchmark criteria.

*p-value <0.05.

After adjustment for platform, content type, commercial intent, and engagement metrics, TikTok platform was independently associated with a lower mDISCERN score compared with Bilibili (*β* = −0.36, *p* = 0.046), while no significant association was observed for GQS or JAMA scores.

Uploader type showed consistent associations across all three quality metrics. Compared with videos uploaded by medical workers, videos from non-medical uploaders were associated with significantly lower mDISCERN, GQS, and JAMA scores (all *p* < 0.001). Videos uploaded by corporate accounts were also associated with lower quality scores across all instruments.

Non-commercial videos were associated with significantly higher mDISCERN and GQS scores compared with commercial videos, but not with JAMA scores. Among engagement indicators, log-transformed likes were negatively associated with mDISCERN and GQS scores, whereas log-transformed favorites showed a positive association with mDISCERN score. Longer video duration was consistently associated with higher quality scores across all three instruments.

### 3.5 Platform-stratified analyses

Platform-specific multivariable regression results are presented in Table 4 and Figure 3. On Bilibili, non-medical uploaders were associated with lower mDISCERN, GQS, and JAMA scores (all *p* < 0.01), while corporate accounts showed no significant association. Non-commercial videos were associated with higher mDISCERN scores. Log-transformed shares were positively associated with mDISCERN (*β* = 0.52, *p* = 0.010) and JAMA scores (*β* = 0.31, *p* = 0.022), whereas log-transformed likes were negatively associated with mDISCERN (*β* = −0.49, *p* = 0.036) and GQS scores (*β* = −0.51, *p* = 0.006). Log-transformed coins were positively associated with GQS scores (*β* = 0.29, *p* = 0.019). Longer time since publication was associated with lower GQS and JAMA scores.

**Table 4:** Multivariable linear regression analysis of factors associated with mDISCERN score, GQS scores and JAMA scores in different platforms.

Platform	Variables	mDISCERN score		GQS scores		JAMA scores	
		*p*	*β* (95%CI)	*p*	*β* (95%CI)	*p*	*β* (95%CI)
Bilibili	Content type						
	Loss weight		Reference		Reference		Reference
	Hypoglycemic	0.282	0.52 (−0.42 ~ 1.47)	0.417	0.31 (−0.44 ~ 1.07)	0.088	0.56 (−0.07 ~ 1.20)
	Loss weight + hypoglycemic	0.507	0.27 (−0.52 ~ 1.06)	0.715	0.12 (−0.51 ~ 0.75)	0.592	0.15 (−0.39 ~ 0.68)
	Others	0.775	−0.11 (−0.86 ~ 0.64)	0.394	−0.26 (−0.85 ~ 0.33)	0.304	0.27 (−0.24 ~ 0.77)
	Uploader type						
	Medical worker		Reference		Reference		Reference
	Non-medical worker	<0.001*	−0.85 (−1.27 ~ −0.42)	<0.001*	−0.94 (−1.28 ~ −0.61)	<0.001*	−1.43 (−1.72 ~ −1.15)
	Corporate account	0.975	−0.02 (−1.27 ~ 1.23)	0.829	−0.11 (−1.10 ~ 0.88)	0.058	−0.83 (−1.67 ~ 0.01)
	Commercial						
	Yes		Reference		Reference		Reference
	No	0.237	0.89 (−0.57 ~ 2.35)	0.303	0.61 (−0.55 ~ 1.77)	0.050*	1.00 (0.02 ~ 1.99)
	Days since publish	0.720	−0.00 (−0.00 ~ 0.00)	0.04*	−0.01 (−0.99 ~ −0.01)	<0.001*	−0.01 (−0.99 ~ −0.01)
	Video duration sec	0.532	0.00 (−0.00 ~ 0.00)	0.300	0.00 (−0.00 ~ 0.00)	0.095	0.00 (−0.00 ~ 0.00)
	Log(Share + 1)	0.010*	0.52 (0.14 ~ 0.91)	0.061	0.30 (−0.01 ~ 0.60)	0.022*	0.31 (0.05 ~ 0.57)
	Log(Favorites + 1)	0.530	0.14 (−0.30 ~ 0.59)	0.751	−0.06 (−0.41 ~ 0.30)	0.487	−0.11 (−0.41 ~ 0.19)
	Log(Likes + 1)	0.036*	−0.49 (−0.95 ~ −0.04)	0.006*	−0.51 (−0.87 ~ −0.15)	0.285	0.17 (−0.14 ~ 0.47)
	Log(Coins + 1)	0.236	0.18 (−0.12 ~ 0.48)	0.019*	0.29 (0.05 ~ 0.53)	0.726	0.04 (−0.17 ~ 0.24)
	Log(Views + 1)	0.173	−0.32 (−0.78 ~ 0.14)	0.824	0.04 (−0.32 ~ 0.40)	0.145	−0.23 (−0.54 ~ 0.08)
	Log(Danmaku + 1)	0.230	0.15 (−0.10 ~ 0.40)	0.514	0.07 (−0.13 ~ 0.26)	0.482	−0.06 (−0.23 ~ 0.11)
Tiktok	Content type						
	Loss weight		Reference		Reference		Reference
	Hypoglycemic	0.942	−0.07 (−1.92 ~ 1.78)	0.818	−0.14 (−1.30 ~ 1.02)	0.807	0.09 (−0.61 ~ 0.79)
	Loss weight + hypoglycemic	0.386	0.35 (−0.44 ~ 1.15)	0.743	−0.08 (−0.58 ~ 0.42)	0.173	0.21 (−0.09 ~ 0.51)
	Others	0.633	−0.15 (−0.78 ~ 0.47)	0.831	0.04 (−0.35 ~ 0.43)	0.321	−0.12 (−0.36 ~ 0.12)
	Uploader type						
	Medical worker		Reference		Reference		Reference
	Non-medical worker	<0.001*	−1.04 (−1.61 ~ −0.46)	0.018*	−0.44 (−0.80 ~ −0.08)	<0.001*	−1.86 (−2.08 ~ −1.65)
	Corporate account	0.049*	−1.02 (−2.03 ~ −0.02)	<0.001*	−1.20 (−1.83 ~ −0.57)	<0.001*	−1.63 (−2.01 ~ −1.25)
	Commercial						
	Yes		Reference		Reference		Reference
	No	0.028*	1.80 (0.21 ~ 3.38)	0.091	0.86 (−0.13 ~ 1.86)	0.088	−0.53 (−1.13 ~ 0.07)
	Days since publish	0.499	0.00 (−0.00 ~ 0.00)	0.003*	0.01 (0.01 ~ 0.01)	<0.001*	−0.01 (−0.99 ~ −0.01)
	Video duration sec	0.086	0.00 (−0.00 ~ 0.00)	0.011*	0.01 (0.01 ~ 0.01)	0.662	−0.00 (−0.00 ~ 0.00)
	Log(Share + 1)	0.179	0.15 (−0.07 ~ 0.37)	0.521	0.04 (−0.09 ~ 0.18)	0.782	−0.01 (−0.09 ~ 0.07)
	Log(Favorites + 1)	0.243	0.20 (−0.14 ~ 0.54)	0.019*	0.26 (0.05 ~ 0.47)	0.681	0.03 (−0.10 ~ 0.16)
	Log(Likes + 1)	0.527	−0.12 (−0.47 ~ 0.24)	0.166	−0.16 (−0.38 ~ 0.06)	0.853	0.01 (−0.12 ~ 0.15)
	Log(Comments + 1)	0.011*	−0.25 (−0.44 ~ −0.06)	0.002*	−0.20 (−0.32 ~ −0.08)	0.645	−0.02 (−0.09 ~ 0.06)

mDISCERN, modified DISCERN instrument, GQS, Global Quality Scale; JAMA, Journal of the American Medical Association benchmark criteria.

*p-value <0.05.

On TikTok, both non-medical uploaders and corporate accounts were associated with lower scores across all three instruments (all *p* < 0.05). Non-commercial videos were associated with higher mDISCERN scores (*β* = 1.80, *p* = 0.028). Log-transformed comments were negatively associated with mDISCERN (*β* = −0.25, *p* = 0.011) and GQS scores (*β* = −0.20, *p* = 0.002), while log-transformed favorites were positively associated with GQS scores (*β* = 0.26, *p* = 0.019). Longer video duration was associated with higher GQS scores, and longer time since publication was associated with lower JAMA scores.

## 4 Discussion

In this cross-sectional study, we systematically evaluated the quality of semaglutide-related video content on Bilibili and TikTok using three validated assessment tools. Across the total sample, videos uploaded by medical workers consistently achieved higher mDISCERN, GQS, and JAMA scores than those uploaded by non-medical workers or corporate accounts, an association that remained significant after adjustment for platform, content type, commercial attributes, engagement indicators, and video characteristics. This persistence indicates that uploader background itself, rather than video popularity, is a substantial independent contributor to information quality, likely reflecting differences in content objectives and professional accountability between medical and non-medical uploaders (15, 16). These findings are consistent with previous research on the critical role of professional background in the reliability and completeness of online medical information, and support practical governance strategies such as credential verification, transparent labeling of uploader qualifications, and greater visibility of evidence-based medical content. Because substantial variation remained across platforms, continuous monitoring of platform-specific health information is warranted to reduce the dissemination of misleading pharmacotherapy information (17–19).

The associations between engagement metrics and information quality varied substantially by platform, highlighting that user interaction does not uniformly reflect informational value. On TikTok, a higher number of comments was independently associated with lower mDISCERN and GQS scores. On Bilibili, a higher number of shares were positively associated with mDISCERN and JAMA scores, whereas a higher number of likes was negatively associated with mDISCERN and GQS scores. One plausible explanation is that comments on TikTok are driven more by emotional salience, controversy, or sensational framing than by informational accuracy; content emphasizing rapid weight loss or dramatic personal experiences can generate extensive commentary while offering only a biased discussion of indications, limitations, and risks. By contrast, sharing behavior on Bilibili may reflect a higher threshold of perceived usefulness or credibility, particularly when content is regarded as sufficiently informative to warrant redistribution, whereas the negative association observed for likes on this platform may reflect emotionally resonant personal-experience content that garners broad approval independent of informational completeness. This divergence suggests that the relationship between popularity and quality is platform-dependent rather than universal, reflecting differences in platform culture, audience expectations, and content recommendation algorithms (20–22). Taken together, these findings indicate that user engagement should not be interpreted as a reliable surrogate for the quality of health information across different short-video platforms. In addition, content type was not independently associated with information quality, suggesting that uploader characteristics and presentation style may exert a stronger influence than the intended therapeutic indication (22, 23).

Several public health implications emerge from these findings. The predominance of non-medical uploaders, particularly on certain platforms, together with their relatively lower quality scores, points to a potential mismatch between content popularity and informational reliability, and the inconsistent relationship between engagement metrics and quality further highlights the limitations of using user-interaction indicators as proxies for trustworthy medical information. Collectively, these results advocate for a coordinated effort among healthcare professionals, public health authorities, and platform regulators to enhance the quality of health information on short-video platforms and ensure that widely disseminated medical content remains accurate, balanced, and evidence-based (24, 25). This is particularly important for semaglutide, which has been widely approved for both glycemic control and weight management in China: inaccuracies or incompleteness in short-video content may generate unrealistic expectations, promote inappropriate self-medication, or drive misuse of prescription drugs. Notably, short videos tend to emphasize weight-loss effects while underrepresenting the drug’s primary indication for diabetes management, potentially distorting public understanding of its therapeutic role (23).

These findings carry practical implications at the clinician, platform, and regulatory levels. Clinicians, academic institutions, and public health organizations should be encouraged to actively participate in short-video content creation, given that professional involvement substantially improves the reliability of health information in social media environments (24) and may help counteract misinformation (26). At the platform level, health communication strategies should be tailored to each ecosystem: on TikTok, stronger associations between engagement metrics and information quality suggest that algorithm-driven popularity may amplify highly engaging but low-quality content, supporting interventions such as improved content moderation, health-information labeling, and collaboration with medical experts (21, 27). At the regulatory level, given the growing role of short-video platforms in disseminating medical information, platform operators should strengthen verification mechanisms for content creators, including credential verification and professional labeling, to help users identify authoritative sources (28, 29), and algorithmic recommendation systems could prioritize content from verified medical professionals or evidence-based sources (30). Regulatory agencies and public health institutions should collaborate with platforms to develop evidence-based guidelines, which—together with the measures above—may help reduce misinformation within the evolving short-video ecosystem (31).

Our findings should be interpreted alongside a growing body of literature examining semaglutide-related content on social media. A recent study by Zeng et al. (32) similarly evaluated semaglutide-related videos on TikTok and Bilibili using bivariate comparisons across uploader and content categories; the present study extends this work through multivariable regression analysis adjusted for multiple confounders simultaneously, together with explicit diagnostic assessment of multicollinearity and model stability. Studies conducted on Western platforms have reported markedly different patterns: Propfe and Seifert (33) found that semaglutide-related content on Instagram and TikTok scored very low on a custom 0–5 information completeness scale (mean 0.22 and 0.72, respectively), with over half of before-and-after videos showing signs of AI manipulation—concerns not assessed in the present Chinese-platform context but warranting future investigation. Yeung et al. (34), analyzing English-language YouTube content using multiple search terms (semaglutide, Ozempic, Wegovy, and Rybelsus), found that longer-form videos and those from verified health-source channels demonstrated higher GQS and Modified DISCERN scores, a pattern conceptually consistent with our finding that medical-professional uploaders produced higher-quality content. Notably, several prior studies (35, 36) employed multiple brand-name search terms in addition to the generic drug name; this represents an important methodological consideration discussed further below.

This study has several limitations. First, its cross-sectional design captures content at a single time point (February 4, 2026) and cannot account for temporal changes in platform algorithms or content trends. Second, only two platforms were included, which may limit generalizability to other social media environments. Third, although validated tools were used, subjective judgment is inherent in scoring systems such as mDISCERN, GQS, and JAMA, despite standardized assessment procedures. Fourth, platform recommendation algorithms may influence which videos appear in search results, potentially introducing selection bias. Fifth, although we used the generic Chinese drug name as our sole search term, a supplementary verification search using the Chinese brand name “诺和泰” revealed platform-specific differences: on Bilibili, brand-name search results were substantially confounded by an unrelated homonymous drama title and celebrity references, yielding largely irrelevant content; on TikTok, brand-name search results closely resembled those obtained using the generic name. This suggests our search strategy was appropriate for both platforms and unlikely to have resulted in substantial omission of relevant content, though future multi-keyword studies should remain attentive to lexical ambiguity, particularly on Bilibili.

## 5 Conclusion

Semaglutide-related health information on Chinese short-video platforms showed relatively high subjective quality and usability as reflected by mDISCERN and GQS scores (median 4.0 of 5 for both), but markedly lower adherence to formal transparency benchmarks as reflected by JAMA scores (median 2 of 4), with substantial variability driven primarily by uploader professional background. Content produced by medical professionals tends to provide more reliable information, whereas engagement metrics do not consistently reflect information quality. Strengthening professional participation and platform-level quality control may help improve the reliability of health information in short-video environments.

## Data Availability

The original contributions presented in the study are included in the article/Supplementary material, further inquiries can be directed to the corresponding authors.

## Footnotes

1
https://www.zstats.net
