# Supplementary material for: Quality and reliability of semaglutide-related health information on Chinese short-video platforms: a cross-sectional study of Bilibili and TikTok
Source: Front Public Health. 2026 Aug 5;14:1826485. doi: 10.3389/fpubh.2026.1826485 (PMC13485863; doi:10.3389/fpubh.2026.1826485)
Supplement: Supplementary file 1 [file Supplementary_file_1.DOCX]

**Supplementary Materials**

*Supplementary Table S4: Multicollinearity (VIF) Analysis*

**Methods note**

To formally assess multicollinearity prior to regression modeling, variance inflation factors (VIF) and tolerance values were calculated separately for each platform, with Platform itself excluded from each within-platform analysis (as it is a constant within a single platform and therefore not meaningfully assessable for collinearity in that context). On Bilibili, 107 videos were available; Comments was entirely missing for this platform (a TikTok-specific metric) and was therefore excluded from the Bilibili collinearity analysis, and a further 16 rows with missing values on other variables were excluded, yielding 91 videos for this analysis. On TikTok, 118 videos were available; Total_Views, Total_Danmaku, and Coins were entirely missing (Bilibili-specific metrics) and were excluded from the TikTok collinearity analysis, and 1 row with a missing content_type value was excluded, yielding 117 videos for this analysis.

Content-attribute variables (content_type, uploader_type, commercial) showed no meaningful collinearity on either platform (all VIF < 1.2; all tolerance > 0.85). In contrast, engagement/popularity metrics showed severe collinearity on Bilibili, where Likes, Favorites, Shares, Total_Views, Total_Danmaku, and Coins all exceeded the conventional VIF threshold of 10 (Coins: VIF = 388.057; Total_Danmaku: VIF = 268.893; Likes: VIF = 236.687; Favorites: VIF = 106.286; Total_Views: VIF = 113.368; Shares: VIF = 46.729), indicating that these indicators largely capture a shared underlying "content popularity" dimension rather than independent informational signals. On TikTok, collinearity among engagement metrics was less severe but still notable: Likes exceeded the conventional threshold (VIF = 29.642), Favorites and Shares approached this threshold (VIF = 8.975 and 8.779, respectively), and Comments remained below the conventional threshold (VIF = 4.344). Days since publication and video duration were not entered into the collinearity assessment alongside engagement metrics, as these structural/temporal variables are conceptually distinct from popularity indicators and were retained as covariates in all regression models without exclusion concerns.

Based on these findings, engagement metrics were entered individually in separate regression models rather than simultaneously, as reported in Methods Section 2.5 and reflected in Table 4 and Supplementary Table S5 (ordinal regression sensitivity analysis).

**Supplementary Table S4. Variance inflation factor (VIF) and tolerance values for content-attribute and engagement variables, by platform.**

| **Variable** | **Bilibili VIF (n=91)** | **Bilibili Tolerance** | **Interpretation** | **TikTok VIF (n=117)** | **TikTok Tolerance** | **Interpretation** |
| --- | --- | --- | --- | --- | --- | --- |
| **content_type** | 1.045 | 0.957 | No collinearity | 1.099 | 0.910 | No collinearity |
| **uploader_type** | 1.114 | 0.897 | No collinearity | 1.113 | 0.899 | No collinearity |
| **commercial** | 1.145 | 0.873 | No collinearity | 1.148 | 0.871 | No collinearity |
| **Likes** | 236.687 | 0.004 | Severe collinearity | 29.642 | 0.034 | Severe collinearity |
| **Favorites** | 106.286 | 0.009 | Severe collinearity | 8.975 | 0.111 | Borderline/elevated |
| **Shares** | 46.729 | 0.021 | Severe collinearity | 8.779 | 0.114 | Borderline/elevated |
| **Comments** | N/A a | N/A a | Not applicable | 4.344 | 0.230 | Below conventional threshold |
| **Total_Views** | 113.368 | 0.009 | Severe collinearity | N/A b | N/A b | Not applicable |
| **Total_Danmaku** | 268.893 | 0.004 | Severe collinearity | N/A b | N/A b | Not applicable |
| **Coins** | 388.057 | 0.003 | Severe collinearity | N/A b | N/A b | Not applicable |
| **Days_Since_Publish** | —c | —c | No collinearity (reference) | —c | —c | No collinearity (reference) |
| **Video_Duration_Sec** | —c | —c | No collinearity (reference) | —c | —c | No collinearity (reference) |

*a Not applicable: Comments was not recorded on Bilibili (a TikTok-specific engagement metric). b Not applicable: Total_Views, Total_Danmaku, and Coins were not recorded on TikTok (Bilibili-specific engagement metrics). c Days_Since_Publish and Video_Duration_Sec were retained as covariates in all regression models; formal VIF values for these two variables were not a focus of this diagnostic, which targeted multicollinearity among engagement/popularity indicators specifically. Conventional interpretation: VIF < 5 (tolerance > 0.20), no meaningful collinearity; VIF 5–10 (tolerance 0.10–0.20), borderline/elevated; VIF > 10 (tolerance < 0.10), severe collinearity.*
