# Supplementary material for: Quality and reliability of semaglutide-related health information on Chinese short-video platforms: a cross-sectional study of Bilibili and TikTok
Source: Front Public Health. 2026 Aug 5;14:1826485. doi: 10.3389/fpubh.2026.1826485 (PMC13485863; doi:10.3389/fpubh.2026.1826485)
Supplement: Supplementary file 2 [file Supplementary_file_2.DOCX]

**Supplementary Materials**

*Sensitivity Analysis: Ordinal Logistic Regression*

**Methods note**

Ordinal logistic regression was performed as a sensitivity analysis to account for the bounded and ordinal nature of the mDISCERN, GQS, and JAMA scores. Given severe multicollinearity among engagement metrics (variance inflation factor [VIF] > 10 for several indicators within each platform), each engagement variable was entered individually in a separate model alongside uploader type, video duration, and days since publication. Because the Corporate account category was sparsely represented on both platforms (n = 2 on Bilibili; n = 5 on TikTok), this category was combined with the Non-medical worker category into a single "Non-medical/Corporate" group to avoid estimation instability arising from complete or quasi-complete separation. All models were fitted using the proportional-odds ordinal logistic regression approach.

On TikTok, the relationship between uploader type and JAMA score exhibited quasi-complete separation: non-medical/corporate uploaders were concentrated almost exclusively at low JAMA scores (0–1), with no overlap with medical-worker uploaders at higher JAMA scores (3–4). This precluded stable coefficient estimation, and results for this platform–outcome combination are therefore not reported in Supplementary Table S4. Findings regarding JAMA scores on TikTok instead rely on the multivariable linear regression and descriptive comparisons reported in the main text (Tables 2 and 4).

**Supplementary Table S5. Ordinal logistic regression of mDISCERN, GQS, and JAMA scores, with engagement metrics entered individually.**

| **Platform** | **Outcome** | **Engagement metric** | **Metric coef. (SE)** | **Metric P** | **Uploader coef. (SE) a** | **Uploader P** | **Note** |
| --- | --- | --- | --- | --- | --- | --- | --- |
| Bilibili | mDISCERN | Likes | -0.026 (SE 0.098) | 0.790 | -2.278 (SE 0.488) | <0.001 |  |
| Bilibili | mDISCERN | Favorites | 0.091 (SE 0.104) | 0.381 | -2.280 (SE 0.485) | <0.001 |  |
| Bilibili | mDISCERN | Shares | 0.086 (SE 0.099) | 0.384 | -2.274 (SE 0.485) | <0.001 |  |
| Bilibili | mDISCERN | Total_Views | -0.064 (SE 0.113) | 0.574 | -2.269 (SE 0.489) | <0.001 |  |
| Bilibili | mDISCERN | Total_Danmaku | -0.014 (SE 0.116) | 0.903 | -2.275 (SE 0.489) | <0.001 |  |
| Bilibili | mDISCERN | Coins | 0.012 (SE 0.122) | 0.922 | -2.282 (SE 0.488) | <0.001 |  |
| Bilibili | GQS | Likes | 0.007 (SE 0.097) | 0.946 | -2.160 (SE 0.448) | <0.001 |  |
| Bilibili | GQS | Favorites | 0.117 (SE 0.103) | 0.256 | -2.185 (SE 0.450) | <0.001 |  |
| Bilibili | GQS | Shares | 0.108 (SE 0.099) | 0.274 | -2.183 (SE 0.450) | <0.001 |  |
| Bilibili | GQS | Total_Views | 0.007 (SE 0.112) | 0.951 | -2.161 (SE 0.451) | <0.001 |  |
| Bilibili | GQS | Total_Danmaku | 0.136 (SE 0.106) | 0.202 | -2.276 (SE 0.461) | <0.001 |  |
| Bilibili | GQS | Coins | 0.146 (SE 0.112) | 0.193 | -2.225 (SE 0.454) | <0.001 |  |
| Bilibili | JAMA | Likes | 0.144 (SE 0.095) | 0.129 | -4.229 (SE 0.606) | <0.001 |  |
| Bilibili | JAMA | Favorites | 0.175 (SE 0.100) | 0.079 | -4.230 (SE 0.605) | <0.001 |  |
| Bilibili | JAMA | Shares | 0.182 (SE 0.099) | 0.067 | -4.242 (SE 0.605) | <0.001 |  |
| Bilibili | JAMA | Total_Views | 0.045 (SE 0.107) | 0.677 | -4.122 (SE 0.600) | <0.001 |  |
| Bilibili | JAMA | Total_Danmaku | 0.120 (SE 0.102) | 0.236 | -4.240 (SE 0.613) | <0.001 |  |
| Bilibili | JAMA | Coins | 0.205 (SE 0.108) | 0.057 | -4.313 (SE 0.615) | <0.001 |  |
| TikTok | mDISCERN | Likes | 0.113 (SE 0.096) | 0.240 | -3.483 (SE 0.512) | <0.001 |  |
| TikTok | mDISCERN | Favorites | 0.126 (SE 0.091) | 0.168 | -3.417 (SE 0.512) | <0.001 |  |
| TikTok | mDISCERN | Shares | 0.149 (SE 0.079) | 0.059 | -3.441 (SE 0.513) | <0.001 |  |
| TikTok | mDISCERN | Comments | 0.030 (SE 0.088) | 0.732 | -3.520 (SE 0.528) | <0.001 |  |
| TikTok | GQS | Likes | -0.087 (SE 0.102) | 0.395 | -3.611 (SE 0.558) | <0.001 |  |
| TikTok | GQS | Favorites | -0.035 (SE 0.094) | 0.707 | -3.617 (SE 0.562) | <0.001 |  |
| TikTok | GQS | Shares | -0.037 (SE 0.081) | 0.645 | -3.619 (SE 0.561) | <0.001 |  |
| TikTok | GQS | Comments | -0.162 (SE 0.096) | 0.090 | -3.406 (SE 0.564) | <0.001 |  |
| TikTok | JAMA | Likes | — | — | — | — | *Not reported b* |
| TikTok | JAMA | Favorites | — | — | — | — | *Not reported b* |
| TikTok | JAMA | Shares | — | — | — | — | *Not reported b* |
| TikTok | JAMA | Comments | — | — | — | — | *Not reported b* |

*a Uploader coefficient reflects Non-medical/Corporate vs. Medical worker (reference). All models additionally adjusted for video duration (seconds) and days since publication. Engagement metrics were log-transformed as log(x + 1) prior to analysis. b Coefficients not reported due to quasi-complete separation between uploader type and JAMA score on TikTok (see Methods note above); SE for the uploader term exceeded 1.9 and the coefficient exceeded 9 in absolute value across all four engagement-metric models, indicating non-identifiable estimates rather than a genuine large effect.*
