# Supplementary material for: Quality and reliability of semaglutide-related health information on Chinese short-video platforms: a cross-sectional study of Bilibili and TikTok
Source: Front Public Health. 2026 Aug 5;14:1826485. doi: 10.3389/fpubh.2026.1826485 (PMC13485863; doi:10.3389/fpubh.2026.1826485)
Supplement: Supplementary file 3 [file Table_1.docx]

**Supplementary Tables**

**Supplementary Table S1. Global Quality Scale (GQS)**

The Global Quality Scale (GQS) is a 5-point Likert scale used to assess the overall quality, flow, and usefulness of health information for patients.

| **Score** | **Description** |
| --- | --- |
| **1** | Poor quality, poor flow, most information missing; not useful for patients |
| **2** | Generally poor quality and limited information; some topics addressed but many important aspects missing |
| **3** | Moderate quality; some important information adequately discussed but others poorly covered |
| **4** | Good quality and generally useful; most relevant information covered |
| **5** | Excellent quality, very useful for patients; comprehensive and well-structured information |
