# Supplementary material for: Quality and reliability of semaglutide-related health information on Chinese short-video platforms: a cross-sectional study of Bilibili and TikTok
Source: Front Public Health. 2026 Aug 5;14:1826485. doi: 10.3389/fpubh.2026.1826485 (PMC13485863; doi:10.3389/fpubh.2026.1826485)
Supplement: Supplementary file 4 [file Table_2.docx]

**Supplementary Table S2. Modified DISCERN (mDISCERN)**

The modified DISCERN instrument evaluates the reliability of health information in online media. Each item is scored **1 (Yes)** or **0 (No)**. The total score ranges from **0–5**, with higher scores indicating greater reliability.

| **Item** | **Question** |
| --- | --- |
| 1 | Are the aims clear and achieved? |
| 2 | Are reliable sources of information used (e.g., publications, medical professionals)? |
| 3 | Is the information presented balanced and unbiased? |
| 4 | Are additional sources of information listed for patient reference? |
| 5 | Are areas of uncertainty mentioned? |
