# Supplementary material for: Quality and reliability of semaglutide-related health information on Chinese short-video platforms: a cross-sectional study of Bilibili and TikTok
Source: Front Public Health. 2026 Aug 5;14:1826485. doi: 10.3389/fpubh.2026.1826485 (PMC13485863; doi:10.3389/fpubh.2026.1826485)
Supplement: Supplementary file 5 [file Table_3.docx]

**Supplementary Table S3. JAMA Benchmark Criteria**

The JAMA benchmark criteria assess the transparency and credibility of online health information sources. Each fulfilled criterion receives **1 point**, with a total score ranging from **0–4**.

| **Criterion** | **Description** |
| --- | --- |
| **Authorship** | Authors and contributors are clearly identified, including credentials and affiliations |
| **Attribution** | References and sources for the content are provided |
| **Disclosure** | Ownership, sponsorship, advertising policies, or conflicts of interest are disclosed |
| **Currency** | Dates of content creation and updates are clearly stated |
